# Radiotherapy Target Volume Definition in Newly Diagnosed High-Grade Glioma Using 18F-FET PET Imaging and Multiparametric MRI: An Inter Observer Agreement Study

**DOI:** 10.3390/tomography8040170

**Published:** 2022-08-16

**Authors:** Brieg Dissaux, Doria Mazouz Fatmi, Julien Ognard, Bastien Allard, Nathalie Keromnes, Amina Latreche, Amandine Lepeuve, Ulrike Schick, Vincent Bourbonne, Douraied Ben Salem, Gurvan Dissaux, Solène Querellou

**Affiliations:** 1 Radiology Department, University Hospital, CHU de la Cavale Blanche, Boulevard Tanguy Prigent, CEDEX, 29609 Brest, France; 2 Groupe d’Étude de la Thrombose Occidentale GETBO (Inserm UMR 1304), Université de Bretagne Occidentale, CHU de la Cavale Blanche, Boulevard Tanguy Prigent, CEDEX, 29609 Brest, France; 3 UFR Médecine, Université de Bretagne Occidentale, 22, rue Camille Desmoulins-CS93837, CEDEX 3, 29238 Brest, France; 4 Laboratoire de Traitement de l’Information Médicale—LaTIM (Inserm UMR 1101), Université de Bretagne Occidentale, 5 Avenue Foch, CEDEX, 29200 Brest, France; 5 Nuclear Medicine Department, University Hospital, CHU de la Cavale Blanche, Boulevard Tanguy Prigent, CEDEX, 29609 Brest, France; 6 Radiation Oncology Department, University Hospital, CHU de la Cavale Blanche, Boulevard Tanguy Prigent, CEDEX, 29609 Brest, France

**Keywords:** inter-observer agreement study, 18-F-FET-PET/CT, high-grade glioma, tumor volume delineation, multiparametric MRI

## Abstract

Background: The aim of this prospective monocentric study was to assess the inter-observer agreement for tumor volume delineations by multiparametric MRI and 18-F-FET-PET/CT in newly diagnosed, untreated high-grade glioma (HGG) patients. Methods: Thirty patients HGG underwent *O*-(2-[18F]-fluoroethyl)-l-tyrosine(18F-FET) positron emission tomography (PET), and multiparametric MRI with computation of rCBV map and K2 map. Three nuclear physicians and three radiologists with different levels of experience delineated the 18-F-FET-PET/CT and 6 MRI sequences, respectively. Spatial similarity (Dice and Jaccard: DSC and JSC) and overlap (Overlap: OV) coefficients were calculated between the readers for each sequence. Results: DSC, JSC, and OV were high for 18F-FET PET/CT, T1-GD, and T2-FLAIR (>0.67). The Spearman correlation coefficient between readers was ≥0.6 for these sequences. Cross-comparison of similarity and overlap parameters showed significant differences for DSC and JSC between 18F-FET PET/CT and T2-FLAIR and for JSC between 18F-FET PET/CT and T1-GD with higher values for 18F-FET PET/CT. No significant difference was found between T1-GD and T2-FLAIR. rCBV, K2, b1000, and ADC showed correlation coefficients between readers <0.6. Conclusion: The interobserver agreements for tumor volume delineations were high for 18-F-FET-PET/CT, T1-GD, and T2-FLAIR. The DWI (b1000, ADC), rCBV, and K2-based sequences, as performed, did not seem sufficiently reproducible to be used in daily practice.

## 1. Introduction

Gliomas are the second most common form of primary brain tumor in adults [1]. In the United States and Europe, the incidence is around 4–5 cases per 100,000 people per year [1]. The first-line treatment in these patients is currently chemoradiotherapy, after most complete surgical resection [1]. Multimodal imaging is thought to be an interesting approach to improve treatment planning for high-precision radiotherapy for patients with high-grade glioma (HGG) [2,3].

Currently, two different classes of radiotracers have been used in neuro-oncology as FluroDeoxyGlucose (18F-FDG) to explore glucose metabolism and amino-acid tracers as 18F-FET. Due to high uptake in the normal brain with a lower signal-to-noise ratio for brain tumors and high uptake in inflammatory lesions, the use of 18F-FDG has decreased. Conversely, the use of radiolabeled amino acids, especially 18F-FET, has grown in recent years. The main advantages of 18F-FET are high in vivo stability and uptake based predominantly on increased transport via the amino acid transport system [4,5]. 18F-FET provides metabolic data for the management of brain tumors with a higher specificity than 18F-FDG for the detection of brain tumors [6]. 

Indeed, according to actual guidelines, radiotherapy target volumes are based on contrast-enhanced (CE) T1-weighted magnetic resonance imaging (MRI) and T2-weighted fluid-attenuated inversion recovery (T2-FLAIR) sequences [2], and recent studies have suggested that the combined use of multiparametric perfusion MRI and O-(2-[18F]-fluoroethyl)-l-tyrosine (18F-FET) positron emission tomography–computed tomography (PET/CT) might be superior to conventional MRI for the better depiction of tumor tissue and extent [2,7,8,9,10]. 

Hutterer et al. reported that 72% of low-grade and HGG lacking contrast enhancement on MRI showed 18F-FET uptake [11]. The Response Assessment in Neuro-Oncology (RANO) working group and the European Association for Neuro-Oncology reported recommendations for the clinical use of PET imaging in gliomas. They reported that in newly diagnosed glioblastoma, metabolically active tumor with 18F-FET PET/CT was larger than contrast enhancement [12]. The joint practice guidelines described common clinical indications for PET imaging in glioma including the definition of the optimal biopsy site and the delineation of tumor extent for surgery and radiotherapy planning [3].

These data could result in a change in radiotherapy volumes to better target tumor infiltration and reduce recurrence and the risk of radionecrosis to surrounding healthy tissues [13,14,15]. Indeed, most patients treated with radiotherapy plus concomitant and adjuvant radiotherapy have central recurrences and 10% of them have new distant lesions that may occur [13]. The use of multiparametric perfusion MRI and 18F-FET could help in better depicting tumor extent or highly metabolic foci in care and thus eventually lead to a more targeted radiation therapy planning. 

These might impact the mortality of these tumors with a poor prognosis, and a median survival of 15–20 months [16]. Due to the therapeutic consequences, especially for radiotherapy planning, the reproducibility of the delineation of tumor volumes is particularly important to assess. Therefore, in this study, we sought to assess the interobserver agreement of multiparametric MRI and 18F-FET PET/CT for the tumor volume delineation in HGG. To the best of our knowledge, no study has compared the observer agreements in this specific indication.

## 2. Materials and Methods

This prospective monocentric study was approved by the institutional review board of the University Hospital of Brest (N°2016. CE14) and registered in ClinicalTrial.gov registry (NCT03370926). Informed consent for study participation was obtained from all patients.

### 2.1. Patient Population

The eligible patients were older than 18 years old, have a histologically proven high-grade glioma (grade 3 or 4 according to 2016 World Health Organization (WHO)), and had an Eastern Cooperative Oncology Group performance status ≤ 2 [17]. The exclusion criteria were a pregnant or breastfeeding woman, contraindications to MRI and/or 18F-FET PET/CT, and a history of encephalic radiotherapy [7].

### 2.2. Imaging Protocol

#### 2.2.1. MRI

MR imaging was performed using a 3T Achieva dStream MRI scanner (Philips Healthcare, Best, Netherlands), a 1.5 T Optima MRI scanner (General Electric Healthcare, Milwaukee, WI, USA), or a 1.5 T Magnetom Avanto Fit (Siemens Healthineers, Erlangen, Germany). 

Briefly, standard imaging included diffusion-weighted imaging (DWI) (b0 and b1000 with apparent diffusion coefficient (ADC) map), T2-weighted fluid-attenuated inversion recovery (T2-FLAIR) sequence, and a 3D-T1-weighted MRI scan after injection (T1-GD) of a standard dose of contrast agent (Gd-DTPA; 0.1 mmol/kg body weight). For perfusion-weighted imaging, dynamic susceptibility-weighted contrast-enhanced T2* (PWI) was achieved. Parametric maps of relative cerebral blood volume corrected for contrast leakage (rCBV) and of a permeability estimation map (K2) were created from PWI using (v3.0 Olea Medical, La Ciotat, France) [18,19].

#### 2.2.2. 18F-FET PET/CT

All patients fasted for at least 4 h before PET/CT, as per the European Association of Nuclear Medicine (EANM) guidelines, for brain tumor imaging using labeled amino acid analogues [3]. PET imaging was performed on a Biograph mCT PET/CT system (Siemens, Siemens Healthineers, Knoxville, TN, USA). For attenuation correction, a low-dose CT scan was performed without iodine contrast. CT acquisition parameters were 16 × 1.2 mm pitch 0.55 with automatic kVp and mAs modulation. CT reconstruction parameters were slice thickness 3/3 mm, convolution kernel H31s, field of view 500 mm for attenuation correction, and slice thickness 2/1.2 mm, convolution kernel J30s, safire 3, and field of view 300 mm for reading. After CT examination, the acquisition was centered on the head and consisted of 40 min dynamic acquisition after the intravenous injection of 3 MBq/kg. PET dynamic reconstructions were performed with 10 × 4 min frames, the reconstruction algorithm was 3DOSEM + TOF + PSF (TrueX) with 200^2^ matrix, zoom2, 2 iterations, 21 subsets, and gaussian post-filter 2 mm. A single static 18F-FET PET/CT frame was obtained by some 20–40 min.

The study stipulated the time between MRI and 18F-FET PET/CT should not exceed 14 days [7].

### 2.3. Target Volume Delineations

In this present study, target volumes were retrospectively assessed independently by three nuclear medicine physicians and three radiologists, respectively. Physicians had different levels of expertise in reading 18F-FET PET/CT and MRI. Nuclear medicine physicians had 19 (SQ: senior1′), 11 (NK: senior2′), and 1 (BA: junior’) of experience, respectively. Radiologists had 9 (JO: senior1), 7 (BD: senior2), and 4 (DM: junior) years of experience respectively.

Data analysis took place from 1 November 2020 to 31 July 2021 to assess interobserver agreement for both imaging modalities (MRI and 18F-FET PET/CT).

This work was performed on MIM Maestro^®^ v7.1.2 (MiM^®^ software Inc., Cleveland, OH 44122, USA). Delineation was blinded to the initial interpretation and any clinically relevant information or imaging results. 

#### 2.3.1. MRI Delineation

All MRI sequences for each patient were analyzed in the following order: T1-GD, T2-FLAIR, rCBV, K2, DWI (b1000), and ADC. On this one hand, the entire lesion had to be segmented, including the centro-tumoral necrosis or hemorrhage areas on morphological sequences such as T1-GD and T2-FLAIR. On the other hand, for functional sequences such as rCBV, K2, DWI (b1000), and ADC, the radiologists had to delineate only the signal abnormalities (hypersignal of neoangiogenesis of the rCBV, K2, and DWI, and hyposignal of ADC). The physicians could use morphological sequences (displayed along) and should not delineate areas that appeared really hemorrhagic.

#### 2.3.2. 18F-FET PET/CT Delineation

The GTV-FET PET was defined by a 3-dimensional automatic segmentation using a tumor-to-brain ratio (TBR) of ≥1.6 within a 30 mm margin around the GTV-MRIc. This threshold is based on a biopsy-controlled study in cerebral gliomas, which demonstrated that a lesion-to-brain ratio of 1.6, best separates tumoral from peritumoral tissue [20]. The normal contralateral uptake (background activity) was defined as an area of normal brain tissue including white and grey matter on the contralateral hemisphere. It was defined by drawing a crescent-shaped volume of interest (VOI) (called “banana”) resulting from the summation of 6 subsequent ROIs 20–25 mm in diameter [16]. For the last step of PET/CT analysis, each observer had to remove uptake related to physiological uptake as skin or vessels. 

### 2.4. Calculation of Spatial Correlation and Overlap between Different Sequences of MRI-Based and 18F-FET PET-Based Tumor Volumes 

As a measure of spatial correlation between MRI-based and PET-based volumes, the Dice Similarity Coefficient (DSC) and the Jaccard Similarity Coefficient (JSC) were calculated [7,8,21,22]. To assess the interobserver agreement in tumor delineation, median Dice coefficient (DC), Jaccard (JC), and Overlap (OV) were calculated over all pairs of observers. Overlap is the volumetric difference between the volumes of interest (VOI) and is defined as the ratio between the intersection and the smallest volume [7,8]. Values range between 0 and 1 and indicate spatial similarity and overlap. Value of 0 indicates no similarity or overlap, whereas a value of 1 indicates perfect agreement [22].

Descriptive statistics were presented as mean and median. The non-parametric Friedman’s repeated measures test and Bonferroni correction for multiple intergroup comparison tests were used. *p* values less than 0.001 were considered significant with 95% confidence intervals (95% CI). Spearman’s correlation coefficient was calculated. Agreements between tumor volumes delineated with each sequence for each reader were calculated through intraclass coefficient correlation. Statistical analysis was performed using the statistical software package Addinsoft, 2018, XLSTAT 2018: Data Analysis and Statistical Solution for Microsoft Excel (Paris, France).

## 3. Results

### Patients

From November 2016 to December 2018, 30 patients (20 male, 10 female) with newly diagnosed HGG (2016 WHO) were prospectively included. Median (range) age was 63 years (24–77) [17]. Twenty-nine out of 30 FET-PET were analyzed. Indeed, the data of one patient were missing due to agent injection issues. Two out of thirty did not have any rCBV and k2 because MR-PWI sequences failed due to an agent injection issue. All other MRI sequences (T1-GD, T2-FLAIR, b1000, and ADC) were available for analysis. Patient and tumor characteristics are described in Table 1.

The median delay between MRI and 18F-FET PET/CT was 6 (1–40) days. The median delay between surgery/biopsy and radiotherapy planning CT was 22 (13–72) days. Twenty-seven patients were scanned using a 1.5T MR scanner and three patients were scanned using a 3T MR scanner. 

Table 2 shows calculation of spatial correlation (DSC and JSC) and overlap for each sequence and for each pair of readers. 18F-FET PET/CT, T1-GD, and T2-FLAIR sequences show higher DCS, JSC, and overlap than PWI (rCBV, K2) and DWI (b1000, ADC)-based sequences.

Table 3 shows the Spearman correlation coefficients between the different pairs of readers for DCS, JSC, and overlap for each sequence. These correlations appear to be strong to very strong for 18F-FET PET/CT, T1-GD, and T2-FLAIR. On the other hand, PWI (rCBV, K2) and DWI (b1000, ADC)-based sequences show very weak to medium correlations.

Table 4 shows cross comparison between DCS, JSC, and overlap for each sequence. Overall, there is no difference between these metrics for T1-GD and T2 FLAIR, whereas there is a difference between 18F-FET PET/CT and T2-FLAIR for DCS and 18F-FET PET/CT and T1-GD and T2-FLAIR for JSC. 

Table 5 summarizes the average volumes delineated for each sequence as well as the intraclass correlation coefficient (ICC) between each reader. 18F-FET PET/CT, T1-GD, and FLAIR sequences show the highest ICC.

**Figure 1:**
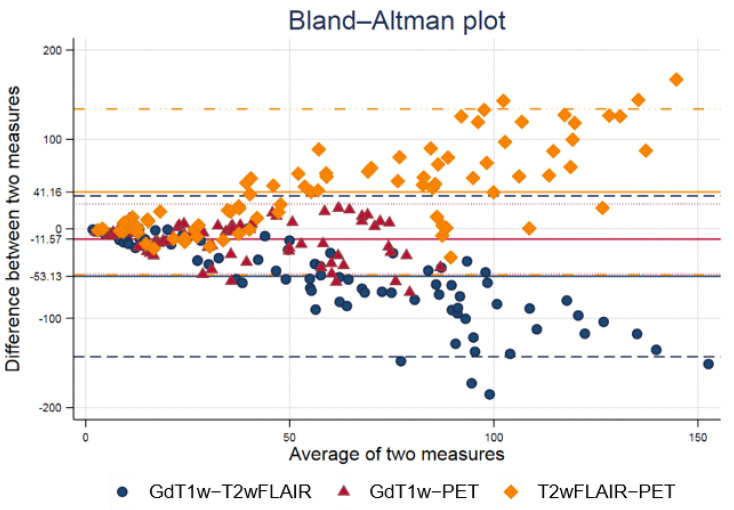
Bland−Altman plot of volumes delineated with 18F-FET PET/CT, T1-GD, and FLAIR sequences. T1-GD and 18F-FET PET/CT volumes are the most similar. T2-FLAIR and T1-GD and T2-FLAIR and 18F-FET volumes PET/CT are the most similar for small volumes, but show higher differences for high volumes.

Figure 1 shows a Bland−Altman plot of volumes delineated with 18F-FET PET/CT, T1-GD, and FLAIR sequences. T1-GD and 18F-FET PET/CT volumes are the most similar. T2-FLAIR and T1-GD and T2-FLAIR and 18F-FET volumes PET/CT are the most similar for small volumes, but show higher differences for high volumes.

**Figure 2:**
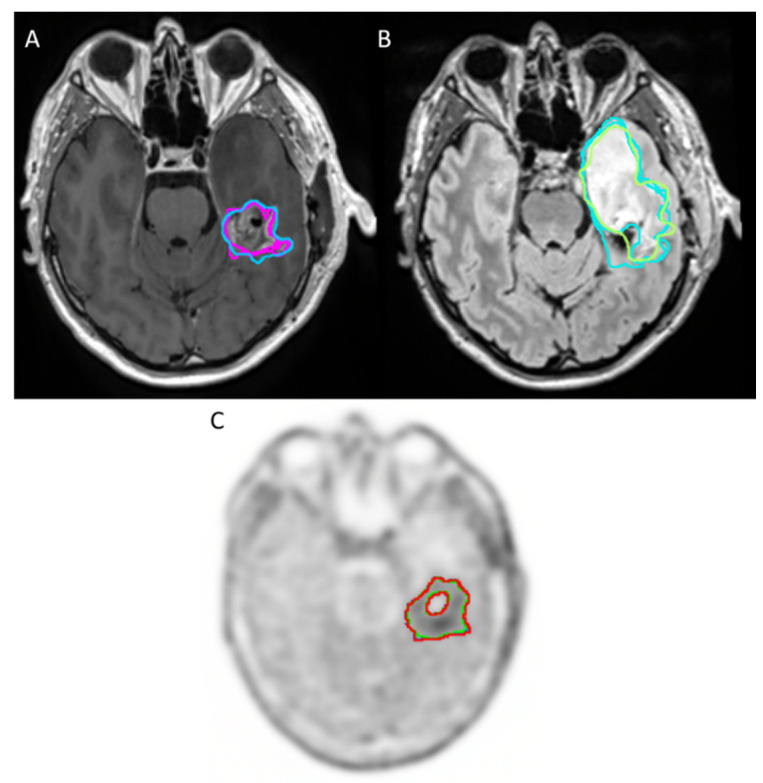
Example of sequences with good agreement for tumor volume delineation in high-grade glioma between readers: (**A**) CE T1-weighted imaging (T1-GD) (**B**) T2-weighted fluid-attenuated inversion recovery (T2-FLAIR), which appears in hypersignal. (**C**) 18F-FET PET/CT.

**Figure 3:**
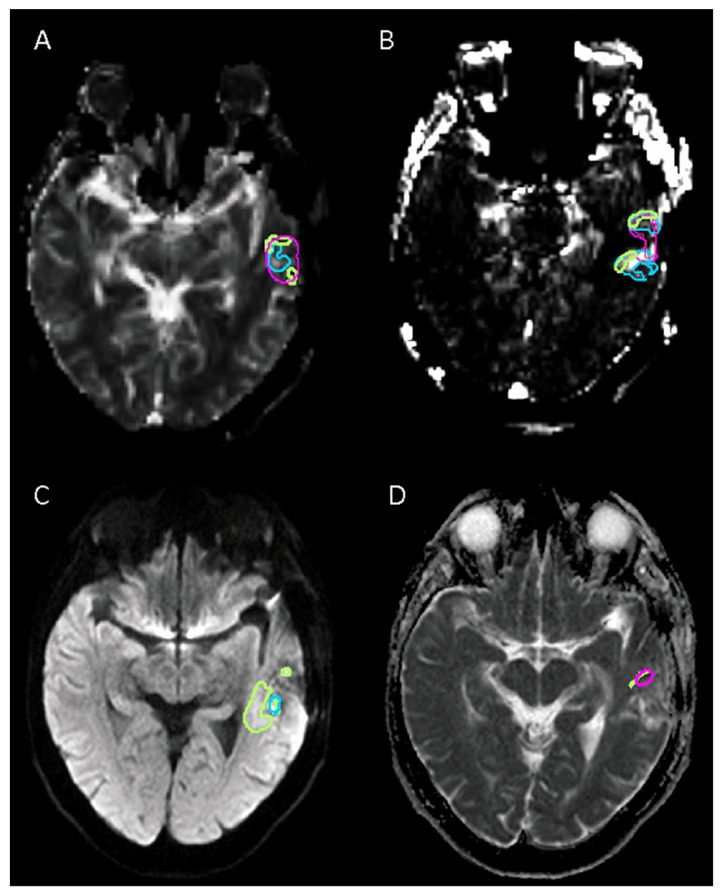
Example of sequences with poor agreement for tumor volume delineation in high-grade glioma between readers: (**A**) Relative cerebral blood volume (rCVB) corrected for contrast leakage shows. (**B**) Permeability estimation map (k2). (**C**) Diffusion-weighted imaging (DWI b1000). (**D**) Apparent diffusion coefficient (ADC) map. In this case, only two readers delineated tumor volume with an ADC map.

Figure 2 and Figure 3 show examples of volumes delineated with the different sequences.

## 4. Discussion

The aim of this work was to assess the interobserver agreements in the delineation of radiotherapy volumes from different imaging sequences (multi-parametric MRI and 18F-FET PET/CT) in high-grade glial lesions 2016 World Health Organization (WHO) grade 3 or 4 [17]. Recent studies have suggested the importance of the combined use of multiparametric perfusion MRI and O-(2-[18F]-fluoroethyl)-l-tyrosine positron emission tomography (18F-FET PET/CT) for the delineation of tumor volumes that can give a better description of the tumor tissue and its extent and could be superior to conventional MRI [7].

The interobserver agreements for the tumor volume delineation in high-grade glioma were high for 18F-FET PET/CT, CE T1-weighted imaging, and T2-FLAIR sequence. The DWI (b1000, ADC) and PWI (rCBV, K2)-based sequences, as performed, did not seem sufficiently reproducible to be used in daily practice. Indeed, our results suggest that lesion volumes defined from 18F-FET PET/CT are the most reproducible, even with a junior nuclear physician (1 year of experience), followed by the morphological MRI sequences CE T1-weighted imaging and the T2-FLAIR sequence. In contrast, lesion volumes defined using functional sequences, such as rCBV or K2 from PWI or ADC and b1000 from DWI, were less reproducible between readers.

A first condition to be met before the use of new imaging sequences for radiotherapy target volume delineation in clinical practice is to ensure their reproducibility. The current guidelines for the definition of the clinical target volume (CTV) are to take a 20 mm margin around the gross tumor volume, which itself is defined by the lesion volume on the T1 sequence, with injection and/or the resection cavity if applicable [2,7]. Stanley et al. studied the impact on dosimetry of interobserver variations in radiotherapy contours of brain metastases. Height physicians delineated fourteen metastases and demonstrated the high degree of interobserver contouring variation and then suggested a consensus prescription to standardize tumor contouring [23]. Kruser et al. reported in their study, the importance of establishing consensus guidelines for CTV delineation in glioblastoma. Ten academic radiation oncologists specializing in brain tumors delineated the CTV of four glioblastoma cases. Initially, moderate to substantial agreement was found on cavity contours plus enhancement (mean kappa of 0.69) and on the T2-FLAIR signal (mean kappa of 0.74). Then, the experts were asked to remove the anatomical barriers while respecting the pathways and, thus, avoiding irradiation of healthy tissues. Then, a very good agreement was found with a kappa ≥0.80 [24].

Our data suggest that the delineation of the tumor volumes using CE T1-weighted imaging and T2-FLAIR sequences that are used in clinical routines are reproducible between readers. Previous studies have focused on the different volumes obtained by delineating high-grade glial lesions using different MRI and nuclear imaging sequences including 18F-FET PET/CT [7,8]. These studies showed differences between these volumes suggesting that they could provide additional information in the study of high-grade glial lesions and particularly the radiotherapy target volume definition [7,8].

We reported in the present work that the delineation of the tumor volumes using 18F-FET PET/CT was reproducible among readers. This supports the hypothesis that this imaging test could be useful for radiotherapy therapeutic planning.

Glial lesions are infiltrative neoplasms, which in response to their need to grow, may develop and recruit blood vessels [25]. In high-grade glioma, this vascular network is often very dense and anarchic with permeable vascular walls. The parameters derived from PWI provide information on tumor vascularization, which reflects tumor invasion, and could allow better tumor delineation [26,27]. They could also allow to distinguish more or less aggressive areas within the tumor volume during radiotherapy treatment [25]. However, our data suggest that without threshold values, the definition of tumor volumes using functional PWI (rCBV, K2) suffers from poor reproducibility.

These differences with 18F-FET PET/CT might be explained by the use of a semi-quantitative threshold for 18F-FET PET/CT. Indeed, this modality has a better signal-to-noise ratio than PWI facilitating this kind of ratio. Smits et al. worked in a multicenter study on the repeatability and reproducibility of rCBV measurements in recurrent glial lesions. They reported significant variability in rCBV measurements and concluded that the different thresholds published in the literature could not be directly applied [28]. DWI sequences (b1000, ADC) have been suggested to precisely delineate the target volume of brain neoplasms and to optimize dose distribution [25,27]. Indeed, they have the ability to explore the heterogeneity of a lesion by assessing the cellular density [25].

Our data suggest that the readers had poor reproducibility in delineating tumor volumes based on DWI sequences. A possible explanation for the poor performance of functional MRI sequences is that they are fast sequences prone to magnetic susceptibility artifacts (hemorrhage, bone, or sinus proximity) and have a low spatial resolution. Li et al. have reported the importance of DWI in radiotherapy planning. However, they reported that the poor reproducibility and the artifacts to which this sequence is subject prevent its generalization. They proposed to associate DWI sequences with high-resolution images after injection or with different b values. Another possible limitation is the lack of a threshold value for ADC delineation. Indeed, ADC is a quantitative value. However, as there is no widely accepted threshold in this indication, we used a manual segmentation. It would be important to investigate whether common training in tumor volume delineation based on functional MRI sequences (PWI, DWI) could improve reproducibility between readers, as proposed by Li et al. [29].

The strengths of this study include a prospective-based well-characterized population with detailed histology. 18F-FET PET/CT and multiparametric MRI were performed within a short delay. The readers’ experiences were varied and balanced for radiologists and nuclear physicians. To our knowledge, there is no other published study that has reported an evaluation of the reproducibility of radiotherapy contours for newly diagnosed high-grade glial lesions with these different MRI sequences and PET-FET.

Nevertheless, our study presents some limitations. First, we selected a small population from only a single center and this may limit the generalizability of the results. Second, multiparametric MRI was performed on different MRI scanners (27/30 patients on a 1.5T). Although this reflects the real-life practice, and each patient has their own comparator for statistical analysis, this may limit the generalizability of the results for 3T MRI. Third, we are aware that there is no commonly accepted threshold for delineating the extent of high-grade glial lesions for rCBV, K2, or ADC, so these sequences were segmented manually, which may have lowered their reproducibility. Fourth, the classification of HGG is based on the WHO 2016. However, this should not influence our results that assess the reproducibility of radiotherapy contours. Fifth, given the study design, this work addresses interobserver variability in the definition of radiotherapy volume, but does not address the possible clinical impact of these results. However, we believe that these data are a prerequisite before testing the hypothesis of a potential clinical impact.

Further studies need to be conducted to test the interobserver reproducibility of CTV based on multiparametric MRI. Indeed, the progress in the treatment of high-grade gliomas with the increase in the dose and the precision of the volume to be irradiated has allowed the secondary effects on the adjacent healthy tissues to be reduced [29]. These imaging sequences might help to improve the accuracy of the target volume to be irradiated while reducing recurrence and radiotoxicity [26].

## 5. Conclusions

In this monocentric inter-observer study, the interobserver agreements for the tumor volume delineation in high-grade glioma were high for 18F-FET PET/CT, CE T1-weighted imaging, and the T2-FLAIR sequence. The DWI (b1000, ADC) and PWI (rCBV, K2)-based sequences, as performed, did not seem sufficiently reproducible to be used in daily practice. Further prospective studies need to be conducted to validate the reproducibility of radiotherapy target volume delineation based on 18F-FET PET/CT imaging and multiparametric MRI before their use in clinical practice.

## Figures and Tables

**Table 1:** Patient and tumor characteristics.

Characteristics	Median/Nb.	Range/Percent
**Age**	63	24–77
**Male**	20	66.7
**Female**	10	33.3
**Histology**		
Grade III	5	16.7
Grade IV	25	83.3
**Multifocal**		
Yes	5	16.7
No	25	83.3
**Extent of resection**		
Biopsy only	14	60
Partial (>5% remaining)	4	10
Subtotal (<5%remaining)	4	13.3
Complete	8	16.7

**Table 2:** Spatial correlation (DSC and JSC) and overlap for each sequence.

Measurement		T1-GD				T2-FLAIR				rCBV				K2				DWI				ADC				PET			
Pair of Reader	Variable	N	Mean	[95% Conf. Interval]		N	Mean	[95% Conf. Interval]		N	Mean	[95% Conf. Interval]		N	Mean	[95% Conf. Interval]		N	Mean	[95% Conf. Interval]		N	Mean	[95% Conf. Interval]		N	Mean	[95% Conf. Interval]	
1	Overlap	30	0.923	0.896	0.950	30	0.852	0.791	0.912	25	0.554	0.439	0.668	27	0.581	0.470	0.691	27	0.482	0.356	0.607	20	0.184	0.042	0.326	29	0.950	0.928	0.973
	Dice	30	0.845	0.804	0.886	30	0.792	0.750	0.833	25	0.416	0.315	0.516	27	0.485	0.379	0.590	27	0.313	0.204	0.423	20	0.090	0.013	0.167	29	0.917	0.892	0.941
	Jacquard	30	0.749	0.693	0.806	30	0.669	0.616	0.723	25	0.293	0.215	0.372	27	0.376	0.291	0.461	27	0.219	0.138	0.299	20	0.056	0.008	0.104	29	0.852	0.812	0.893
2	Overlap	30	0.924	0.903	0.945	30	0.890	0.849	0.930	25	0.458	0.323	0.592	27	0.764	0.674	0.854	27	0.575	0.426	0.723	24	0.159	0.024	0.293	29	0.970	0.958	0.982
	Dice	30	0.863	0.828	0.898	30	0.794	0.740	0.848	25	0.305	0.194	0.416	27	0.595	0.521	0.669	27	0.388	0.258	0.518	24	0.083	0.000	0.171	29	0.930	0.914	0.947
	Jacquard	30	0.768	0.718	0.817	30	0.680	0.617	0.743	25	0.211	0.128	0.295	27	0.446	0.376	0.517	27	0.295	0.189	0.401	24	0.059	0.000	0.124	29	0.873	0.845	0.900
3	Overlap	30	0.919	0.894	0.944	30	0.886	0.846	0.925	23	0.438	0.300	0.576	27	0.706	0.590	0.821	25	0.430	0.286	0.575	25	0.159	0.044	0.275	29	0.956	0.936	0.976
	Dice	30	0.816	0.767	0.865	30	0.778	0.724	0.832	23	0.298	0.186	0.410	27	0.481	0.389	0.573	25	0.327	0.214	0.440	25	0.085	0.017	0.153	29	0.918	0.895	0.942
	Jacquard	30	0.708	0.644	0.772	30	0.657	0.594	0.719	23	0.205	0.120	0.289	27	0.344	0.268	0.419	25	0.227	0.142	0.313	25	0.056	0.007	0.105	29	0.855	0.816	0.893
All	Overlap	90	0.922	0.908	0.935	90	0.876	0.849	0.903	73	0.484	0.413	0.556	81	0.683	0.623	0.744	79	0.497	0.419	0.575	69	0.166	0.095	0.237	87	0.959	0.948	0.969
	Dice	90	0.841	0.818	0.865	90	0.788	0.760	0.816	73	0.341	0.280	0.401	81	0.520	0.468	0.572	79	0.343	0.278	0.409	69	0.086	0.043	0.129	87	0.922	0.910	0.934
	Jacquard	90	0.742	0.710	0.774	90	0.669	0.635	0.702	73	0.237	0.191	0.284	81	0.389	0.345	0.433	79	0.247	0.196	0.299	69	0.057	0.027	0.087	87	0.860	0.840	0.880

18F-FET PET/CT, T1-GD, and T2-FLAIR sequences show higher DCS, JSC, and overlap than PWI (rCBV, K2) and DWI (B1000, ADC)-based sequences. 18F-FET PET/CT pair of reader 1 senior1′/junior’, 2 senior1′/senior2′ and 3 junior’/senior2′. MRI pair of reader 1 senior1/junior, 2 senior2/junior, and 3 senior1/senior2.

**Table 3:** The correlations between the different pairs of readers for DCS, JSC, and overlap for each sequence. Spearman’s correlation coefficient.

Measurement		T1-GD		T2-FLAIR		rCBV		K2		DWI		ADC		18F-FET PET/CT	
Pair of Readers	Variable	N	Corr.	N	Corr.	N	Corr.	N	Corr.	N	Corr.	N	Corr.	N	Corr.
1–2	Overlap	30	0.062	30	0.110	25	0.068	27	0.441	25	0.306	19	0.213	29	0.706
	Dice	30	0.654	30	0.685	25	0.609	27	0.519	25	0.573	19	0.549	29	0.743
	Jacquard	30	0.536	30	0.688	25	0.576	27	0.598	25	0.573	19	0.549	29	0.740
1–3	Overlap	30	0.793	30	0.572	23	0.399	27	0.551	23	0.786	19	0.036	29	0.748
	Dice	30	0.823	30	0.798	23	0.452	27	0.577	23	0.839	19	0.244	29	0.786
	Jacquard	30	0.713	30	0.812	23	0.414	27	0.669	23	0.843	19	0.228	29	0.787
2–3	Overlap	30	0.285	30	0.243	23	0.376	27	0.609	24	0.469	23	0.382	29	0.568
	Dice	30	0.810	30	0.660	23	0.821	27	0.665	24	0.648	23	0.449	29	0.75
	Jacquard	30	0.810	30	0.681	23	0.819	27	0.668	24	0.644	23	0.440	29	0.743

Dichotomized interpretation: strong to very strong correlation (0.6–1) in blue highlight and very weak to medium (0–0.59) in red highlight. The correlations between the different pairs of readers appear to be strong to very strong for 18F-FET PET/CT, T1-GD, and T2-FLAIR. On the other hand, PWI (rCBV, K2) and DWI (B1000, ADC)-based sequences show very weak to medium correlation. 18F-FET PET/CT pair of reader 1 senior1′/junior’, 2 senior1′/senior2′ and 3 senior2′/junior’. MRI pair of reader 1 junior/senior2, 2 junior/senior2 and 3 senior1/senior2. *Dichotomized Interpretation: Strong to Very strong 0.6–1* 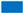
*; Very weak to medium 0–0.59* 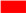.

**Table 4:** Cross comparison between DCS, JSC, and overlap for each sequence.

Cross Comparison		T1-GD		T2-FLAIR	
Variable	Sequence	Mean Diff.	*p*-Value	Mean Diff.	*p*-Value
**Overlap**	T2-FLAIR	−0.046	1.000		
	18F-FET PET/CT	0.037	1.000	0.083	0.37
**Dice**	T2-FLAIR	−0.054	1.000		
	18F-FET PET/CT	0.081	0.124	0.134	<0.001
**Jacquard**	T2-FLAIR	−0.073	0.084		
	18F-FET PET/CT	0.118	<0.001	0.191	<0.001

Overall there is no difference between DCS, JCS, and overlap metrics for T1-GD and T2 FLAIR, whereas there is a difference between 18F-FET PET/CT and T2-FLAIR for DCS and 18F-FET PET/CT and T1-GD and T2-FLAIR for JSC.

**Table 5:** The average volumes delineated for each sequence and the ICC between each reader.

Volume		Reader 1 (cc)			Reader 2 (cc)			Reader 3 (cc)			All (cc)			All		
Sequences	N	Mean	[95% Conf. Interval]		Mean	[95% Conf. Interval]		Mean	[95% Conf. Interval]		Mean	[95% Conf. Interval]		ICC	[95% Conf. Interval]	
T1-GD	30	28.720	19.929	37.511	30.524	21.591	39.458	29.913	20.992	38.833	29.719	24.790	34.647	0.969	0.944	0.984
T2-FLAIR	30	81.105	58.774	103.436	89.579	66.125	113.033	77.868	55.722	100.013	82.850	70.245	95.454	0.929	0.871	0.964
rCBV	28	16.494	9.667	23.322	13.334	6.695	19.972	11.141	4.573	17.709	13.656	9.932	17.380	0.838	0.715	0.916
K2	28	17.223	11.007	23.439	15.037	8.652	21.422	25.955	15.619	36.291	19.405	14.928	23.881	0.748	0.531	0.874
DWI	30	11.189	6.455	15.922	8.919	4.811	13.027	13.544	7.174	19.913	11.217	8.328	14.105	0.596	0.398	0.762
ADC	30	0.803	0.212	1.394	3.848	0.000	8.639	3.248	1.021	5.476	2.633	0.908	4.357	−0.009	−0.184	0.232
18F-FET PET/CT	29	40.537	29.641	51.434	39.549	29.269	49.829	40.743	30.287	51.198	40.276	34.434	46.117	0.986	0.975	0.993

18F-FET PET/CT, T1-GD, and FLAIR sequences show the highest volumes and the highest ICC between readers. 18F-FET PET/CT; reader 1 = senior1′, reader 2 = junior’, reader 3 = senior2′ and MRI; reader 1 = junior, reader 2 = senior1, reader 3 = senior 2.

## Data Availability

Restrictions apply to the availability of these data. Data are available on reasonable request from the authors with the permission of the local Ethic Committee.
